# Systematic Review of the Prevalence and Incidence of Parkinson’s Disease in Asia

**DOI:** 10.2188/jea.JE20081034

**Published:** 2009-11-05

**Authors:** Weerasak Muangpaisan, Hiroyuki Hori, Carol Brayne

**Affiliations:** 1 Department of Preventive and Social Medicine, Faculty of Medicine Siriraj Hospital, Mahidol University, Bangkok, Thailand; 2 Health Policy Bureau, Ministry of Health, Labour and Welfare, Tokyo, Japan; 3 Department of Public Health and Primary Care, University of Cambridge, Cambridge, UK

**Keywords:** epidemiology, prevalence, incidence, Parkinson’s disease, Asia

## Abstract

**Background:**

Parkinson’s disease (PD) is a common neurodegenerative disorder in older people, and half of the world’s older population lives in Asia. However, the epidemiology of PD in Asian countries is poorly understood. This review assembles evidence on the prevalence and incidence of PD in Asian countries and identifies gaps in our present knowledge.

**Methods:**

A systematic search of studies published from 1965 to October 2008 was conducted using MEDLINE and EMBASE. The selection criteria were defined a priori. Prevalence and incidence were standardized to the WHO World Standard Population 2000. Twenty-one original studies were selected for the review. Two studies that described the ethnic origin of participants and contained Asian populations were also included in the analysis.

**Results:**

Excluding one study with questionably low prevalence and incidence, the remaining studies reported a standardized all-age prevalence of 51.3 to 176.9 per 100 000 in door-to-door surveys; prevalence in record-based studies ranged from 35.8 to 68.3 per 100 000. The standardized incidence rates were 8.7 per 100 000 person-years in door-to-door surveys and 6.7 to 8.3 per 100 000 person-years in record-based surveys.

**Conclusions:**

The prevalence of PD in Asian countries was slightly lower than that in Western countries. However, comparison of incidence was difficult because of the small number of studies. Varying methodologies, diagnostic criteria, and case-finding strategies contributed to the considerable variation in the reported prevalence and incidence of PD.

## INTRODUCTION

The age structure of the population worldwide is changing. Less-developed countries are undergoing a demographic transition to aging societies faster than was historically the case for developed countries.^1^ Asia includes a number of less-developed countries in which life expectancy is increasing rapidly. More than 385.4 million people in Asia are 60 years or older and more than 41.9 million people are 80 years or older.^1^ These figures account for approximately 54.7% and 44.5% of the world population in these age groups. In 2007, 40% of the world population lived in China or India. An additional 8 countries account for a further 20% of the world population—4 of which are in Asia (Indonesia, Pakistan, Bangladesh, and Japan).^1^ As a result of this growing older population, diseases and disorders of old age, especially chronic diseases, are a major concern.

Parkinson’s disease (PD) is the second most common neurodegenerative disorder. It is characterized not only by movement abnormalities, but also by nonmotor symptoms such as dementia, depression, visual hallucinations, and autonomic dysfunction. These symptoms cause disability and reduce quality of life. PD also places a heavy burden on caregivers. Because of the global impact of PD, many epidemiological studies have been conducted worldwide over the past few decades. Despite the faster population growth rate and the presence of half the world’s aging population in Asia, few epidemiological studies of PD have been conducted there. Existing reviews of the epidemiology of PD have not focused on Asian countries and have included only a limited number of studies from Asia.^2^^,^^3^ Genetic susceptibility and environmental factors, combined with the effects of aging, play major roles in the etiology of PD. Asian and non-Asian populations have different genetic backgrounds and environmental exposures, which might influence their risk for PD. The objective of this review was to systematically investigate the prevalence and incidence of PD in Asian countries and to examine the underlying problems that affect epidemiological studies of PD in these settings.

## METHODS

### Literature search for Asian studies

A literature search was conducted on the Medline and EMBASE databases for studies investigating the prevalence and incidence of PD. The MeSH keywords “Parkinson”, “Parkinson’s disease”, “Epidemiology”, “Incidence”, “Prevalence” and “Asia” were used. Search limits included Human, All adults, and publication date from 1965 through October 2008. A search of citations of included papers and relevant published reviews was also performed. No language restriction was employed.

Articles were included if the studied populations were larger than 3000 participants in population-based survey studies, unless they were the only study of a country. For record-based studies, a denominator higher than 10 000 participants was required to ensure that the number of cases was sufficient and that the estimates were statistically precise. Because the choice of diagnostic criteria can affect estimates of prevalence and incidence, only articles that defined the diagnostic criteria for PD were included.

The primary search of both databases yielded 251 results. After screening the titles and abstracts, 30 relevant abstracts were selected. Of these, 1 article was deemed irrelevant^4^ and 3 were duplicates.^5^^–^^7^ Two papers were excluded because the studied populations were smaller than specified in the predefined criteria^8^^,^^9^; 2 were excluded because diagnostic criteria were not mentioned.^10^^,^^11^ One study was excluded because the study population was a group of elderly individuals in a home care setting, which was not representative of the general population.^12^ Ultimately, 21 original research studies were selected for the review. Two of these that classified the participants by ethnic origin and included an Asian population were included in the analysis.^13^^–^^15^

### Literature search for worldwide studies

A literature search was conducted on Medline and EMBASE for studies worldwide investigating the prevalence and incidence of PD. The MeSH keywords used for the search were “Parkinson”, “Parkinson’s disease”, “Epidemiology”, “Incidence” and “Prevalence”. Search limits included Human, All adults, English, and publication from 1965 through October 2008. A search of the citations of included papers and published relevant reviews was also performed. One problem with conducting a literature search on worldwide PD studies was that there were many such articles, due to the long history of PD research. Strict inclusion criteria were therefore applied to ensure that the articles retrieved would be of a high standard. The general inclusion criteria were: (1) publication in English, (2) study population larger than 3000 participants for population-based survey studies, unless they were the only study of a country (for registry-based studies, a denominator more than 20 000 participants was required to ensure that the number of cases was sufficient and that the estimates were statistically precise), (3) the diagnostic criteria used for case ascertainment were explicit, (4) the study was not an Asian PD study, and (5) the age-specific rate was reported, to ensure that the prevalence and incidence rates could be compared to those reported in Asian studies.

The primary search of both databases yielded 2817 results. After screening topics and abstracts, 120 relevant abstracts were tagged and saved for more thorough perusal. Ultimately, 32 papers were selected for review after applying the prespecified inclusion criteria. Appendices 1 and 2 (available as Supplementary Data on the journal's website) summarize the characteristics of the selected studies. Appendices 3 and 4 show the reported prevalence and incidence rates.

### Statistical analysis

The major difficulty in determining and reporting the frequency of PD is properly distinguishing between crude, specific, and standardized rates. The crude rate may differ in different populations simply because the demographic structure of the population differs, not because of any actual differences in disease rates. The crude rate is thus only useful for establishing the magnitude of the clinical problem. Age is the strongest risk factor for PD and gender may also contribute to differences in disease frequency. Although PD may be a common neurological disease in a population, disease occurrence may not be common enough to use rates that are age- and gender-specific. Therefore, standardized rates are used in such circumstances. To eliminate the possibility that differences in prevalence and incidence rates across countries could have been due to differences in the age structure of the different countries, we used the WHO World Standard Population 2000 as the standard population. All crude age-specific rates were applied to this standard population for the comparison. The reported 95% confidence intervals for the male:female ratio of PD incidence were retrieved from the included studies and summarized.

## RESULTS

### Description of studies

Of the 21 studies included in this review, 19 reported prevalence rates and 7 (5 of which overlapped with the prevalence studies) reported incidence rates. The studies were conducted in China, Taiwan, Japan, Singapore, India, Israel, and Saudi Arabia. Six studies were conducted in China^16^^–^^21^ and 5 were conducted in Japan.^22^^–^^26^ In the prevalence studies, 11 were conducted by door-to-door survey,^16^^–^^19^^,^^21^^,^^27^^–^^32^ and most of these were conducted in 2 phases: interview screening followed by a neurological examination to confirm a positive result on screening. One prevalence study was a single-phase door-to-door survey by neurologists^17^ and another was a 3-phase survey starting with an interview, which was followed by an examination of participants by medical specialists and confirmation by movement-disorder specialists.^32^ Regarding the incidence studies, 2 were door-to-door surveys^16^^,^^31^ and 5 were record-based surveys.^22^^,^^24^^,^^25^^,^^33^^,^^34^ The age cut-off points for participants varied from none (all ages included) to age older than 55 years. For the purpose of comparison, studies were categorized into screening-based or record-based studies. Factors which could affect the accuracy of data, ie, diagnostic criteria, proportion of cases examined by specialists, and participation rate, are summarized in Tables 1 and 2. The percentage of diagnoses made by specialists ranged from 7% to 100%. The participation rate varied between 57% and 100% in prevalence studies and between 67% and 99% in incidence studies. Four prevalence studies^16^^,^^25^^,^^29^^,^^30^ and 3 incidence studies^16^^,^^20^^,^^22^^,^^25^^,^^35^ did not report participation rates. In most studies, a diagnosis of PD was based on the presence of at least 2 of the 4 cardinal signs. Exclusion of secondary causes of parkinsonism was a frequent criterion in earlier studies; stricter criteria (eg, UKPDS Brain Bank Criteria) were used in later studies.

**Table 1.** Summary of studies on the prevalence of Parkinson’s disease in Asia

Authors/year	Location	Study period	Design	Case-finding strategy	Diagnostic criteria	Age of cases	Percentage examined by specialists	Population	PD cases	Participation rate
Li et al, 1985^21^	China	1983	Door-to-door survey	2-phase door-to-door survey: questionnaire and brief examination; if positive, examined by neurologist	Resting tremor and rigidity and/or hypokinesia and exclusion of other causes	All ages	100%	63 195	28	100%

Bharucha et al, 1993^27^	Bombay, India	1985	Door-to-door survey	2-phase door-to-door survey: questionnaire and neurological examination	≥3 cardinal signs; exclusion of secondary causes	All ages	100%	14 010	46	95%

Wang et al, 1991^16^	China	1986	Door-to-door survey	2-phase door-to-door survey: questionnaire and neurological examination	Schoenberg’s criteria^a^	All ages	100%	3 869 162	566	Not stated

Al Rajeh et al, 1993^28^	Thugbah, Saudi Arabia	1989	Door-to-door survey	2-phase door-to-door survey: questionnaire and neurological examination	≥2 of resting tremor, bradykinesia, rigidity; exclusion of other causes	All ages	100%	22 630	6	100%

Das et al, 1996^29^	West Bengal, India	1989–90	Door-to-door survey	2-phase door-to-door survey: questionnaire and neurological examination	≥2 of 4 cardinal signs; exclusion of drug-induced parkinsonism	All ages	100%	37 286	6	Not stated

Saha et al, 2003^30^	West Bengal, India	1992–3	Door-to-door survey	2-phase door-to-door survey: questionnaire and neurological examination	≥2 of 4 cardinal signs; exclusion of drug-induced parkinsonism	All ages	100%	20 842	Not reported	Not stated

Wang et al, 1996^17^	Kinmen, China	1993	Door-to-door survey	Single-phase door-to-door survey by neurologists	≥2 of 4 cardinal signs, if not receiving antiparkinsonian drugs; Or, ≥1 of 4 cardinal signs if improved by medications	≥50	100%	3915	23	96%

Zhang et al, 2003^18^	Greater Beijing, China	1996–1997	Door-to-door survey with reexamination 2–54 months later	2-phase door-to-door survey: questionnaire and neurological examination	≥3 cardinal signs, or ≥2 cardinal signs if there is ≥1 additional condition: asymmetry, one sign was either resting tremor or bradykinesia, or no levodopa unresponsiveness	≥55	Examined by neurologist, actual number not stated	5743	64	96%

Zhang et al, 2005^19^	Beijing, Xian, Shanghai, China	1997–1998	Door-to-door survey with reexamination in 2 months	2-phase door-to-door survey: questionnaire and neurological examination	≥3 cardinal signs, or ≥2 cardinal signs if there is ≥1 additional conditions: asymmetry, one sign was resting tremor or bradykinesia, or no levodopa unresponsiveness	≥55	100%	29 454	277	73–100%

Chen et al, 2001^31^	Taiwan	1993–1999	Door-to-door survey with follow-up at 7 years	2-phase door-to-door survey: questionnaire and examination by neurologists	≥2 cardinal signs and exclusion of other causes	≥40	100%	75 579	37	88%

Zhang et al, 2005^20^	Linxian, China	1999–2000	Screening of survivors of a cohort study	2 phases: screening with interview and brief neurological examination; then, full neurological examination	UKPDS Brain Bank Criteria	≥50	100%	16 488	86 (plus 203 probable PD and 175 possible PD)	75%

Seo et al, 2007^35^	Korea	1999–2001	Random sample from one cohort study	2 phases: questionnaire and examination by neurologists	UKPDS Brain Bank Criteria	All ages	100%	4700	16	90%

Tan et al, 2004^32^	Singapore	2001–2003	Door-to-door survey of Chinese, Malays, and Indians	3 phases: door-to-door survey, 1. questionnaire 2. examined by medical specialists 3. confirmed by movement- disorder specialists	National Institute of Neurologic Disorders and Stroke Criteria	>50	100%	15 000	46	67%

Harada et al, 1983^22^	Yonago, Japan	1975–1981	Record-based survey	Medical records from hospitals, general practitioners, national health insurance	≥2 of 4 cardinal signs; exclusion of secondary causes	Not clear, likely all ages	45%	125 291	101 (in 5 yrs)	100% in the questionnaire response

Okada et al, 1990^23^	Izumo, Japan	Not stated	Record-based (hospital survey) or door-to-door survey	2 phases: questionnaire and then acquisition of hospital surveys or door-to-door survey	≥2 cardinal signs	≥20	7%	56 689	66	62%

Kusumi et al, 1996^24^	Yonago, Japan	1992–4	Record-based survey	Review of medical records and questionnaires sent to hospitals and clinics	≥2 of 4 cardinal signs, improvement by L-dopa and exclusion of secondary causes	All ages	Not stated	1 342 315	156	99% in the questionnaire response

Moriwaka et al, 1996^25^	Hokkaido, Japan	1992–4	Record-based from neurologists, general hospitals, and Ministry of Welfare and Health	2-phase survey: prevalence survey of entire Hokkaido population then prevalence and incidence in Iwamizawa City 1 year later	≥2 of 4 cardinal signs, negative brain CT scan, response to antiparkinsonian drugs and exclusion of secondary causes	All ages	Not clear (≥49%)	5 643 647 (island of Hokkaido)	5342	Not stated

Anca et al, 2002^40^	Israel	1998	Record-based survey (to medical clinics)	Two questionnaires, then confirmed by neurologist	≥2 of resting tremor, bradykinesia, rigidity, and exclusion of other causes	All ages	100%	73 767	180	57%

Kimura et al, 2002^26^	Japan	2000	Record-based survey in hospitals and clinics	2-step questionnaires	All 5 items: 1. insidious onset after age 20 years 2. resting tremor or cogwheel rigidity with akinesia or small-step gait 3. improvement by antiparkinsonian drugs 4. no history of drug-induced parkinsonism 5. exclusion of other causes	All ages	62%	1 244 040	963	73%

^a^Schoenberg’s criteria: (a) insidiously progressive rest tremor, rigidity, hypokinesia + no definite cause + middle age, (b) diagnosis by neurologist or senior doctor, and (c) exclusion of secondary causes.

**Table 2.** Summary of studies on the incidence of Parkinson’s disease in Asia

Authors/year	Study	Year	Study design	Case findings	Diagnostic criteria	Age of cases	Proportion examined by neurologists	Population	PD cases	Participation rate
Wang et al, 1991^16^	China	1986	Door-to-door survey	2-phase door-to-door survey: questionnaire and neurological examination	Schoenberg’s criteria^a^	All ages	100%	3 869 162	566	Not stated

Chen et al, 2001^31^	Taiwan	1993–9	Door-to-door survey with follow-up at 7 years	2-phase door-to-door survey: ​ - Questionnaire ​ - Examination by neurologists	≥2 cardinal signs and exclude other causes	≥40	100%	75 579	15	88%

Harada et al, 1983^22^	Yonago, Japan	1975–1981	Record-based survey	Medical records from hospitals, general practitioners, national health insurance	≥2 of 4 cardinal signs and exclude secondary causes	Not clear, likely all ages	45%	125 291	10–14 per year	Not stated

Kusumi et al, 1996^24^	Yonago, Japan	1992–4	Record-based survey	Review of medical records and questionnaire sent to hospitals and clinics	≥2 of 4 cardinal signs, improvement by L-dopa and exclude secondary causes	All ages	Not stated	1 342 315	14–24 per year in 4 years	99% in the questionnaire response

Moriwaka et al, 1996^25^	Hokkaido, Japan	1992–4	Record-based from neurologists, general hospitals, and Ministry of Welfare and Health	2-phase survey: prevalence survey of all of Hokkaido, then prevalence and incidence study in Iwamizawa City 1 year later	≥2 of 4 cardinal signs, negative CT brain, response to antiparkinsonian drugs and excluded secondary causes	All ages	Not clear (≥49%)	80 417 (Iwamizawa city)	7	Not stated

Morioka et al, 2002^33^	Wakayama, Japan	1998	Record-based survey	Mailed questionnaire on newly diagnosed cases to clinics and hospitals	1. unclear onset, slow progressive 2. at least 1 of ​ a) tremor ​ b) cogwheel rigidity, akinesia and/or ​ ​ festinating gait ​ c) if a & b are absent, 3 of postural ​ tremor, lead-pipe rigidity, ​ akinesia, festinating gait, and ​ retropulsion are present 3. L-dopa responsive 4. exclude drug-induced disorder and other secondary causes	≥40 years	Not stated	1 372 781	232	99%

Tan et al, 2007^34^	Singapore	2001–3	Record-based + phone interview	Door-to-door survey of prevalence; then phone interview, medical record review, and database merge for incident cases	Gelb criteria	≥50	78%	14 835	12	67%

^a^Schoenberg’s criteria: (a) insidiously progressive rest tremor, rigidity, hypokinesia + no definite cause + middle age, (b) diagnosis by neurologist or senior doctor, and (c) exclusion of secondary causes.

### Measurement of disease frequency

#### Prevalence

Screening for PD by means of door-to-door surveys revealed that 14% to 78% of respondents that were found to have PD had never received a diagnosis of PD. The crude prevalence, age, and sex-specific prevalence are summarized in Table 3. Since prevalence depends on the case-finding strategies employed, it is presented separately in Table 4. Overall, the age-standardized prevalence per 100 000 for all age groups in door-to-door surveys ranged from 16.7 to 176.9, which was higher than the prevalence found in record-based studies—35.8 to 68.3 (Table 4). However, the prevalence reported in 1 study in China^16^ was considerably lower than that noted in other countries, and was lower even than the prevalences reported in other studies conducted in China.^17^^–^^21^ When this study was excluded, the age-standardized prevalence per 100 000 for all age groups in door-to-door surveys ranged from 51.3 to 176.9. There was no striking geographical variation.

**Table 3.** Reported prevalence of Parkinson’s disease in Asia (per 100 000 population)

Studies	%previously undiagnosed	Age at onset	Crude prevalence^a^	Age-specific prevalence^a^
Six cities, China (Li et al, 1985)^21^	Not stated	63.3	Crude rate (all ages): 44 Crude rate (≥50 years): 198 Age-adjusted rate (all ages): 57 (standardized to 1960 US population)	<50: 0 50–59: 92 60–69: 145 ≥70: 615

Bombay, India (Bharucha et al, 1993)^27^	Not stated	Not stated	Crude rate (all ages): 328.3 Age-adjusted rate (all ages): M 234.8, F 153.8, B 192	35–39: M 218.3, F 0, B 103.8 50–59: M 204.3, F 80.2, B 134.8 60–69: M 608.5, F 555.6, B 580.8 70–79: M 1899.8, F 935.8, B 1356.4 80–89: M 1718.2, F 2215.1, B 1976.9

29 provinces, China (Wang et al, 1991)^16^	Not stated	40–89	Crude rate (all ages): M 16.9, F 12.4, B 14.6 Age-adjusted rate (all ages): 10.8 (standardized to 1982 Chinese population) Age-adjusted rate (all ages): 14.9 (standardized to 1973–7 World population)	0–49: M 1, F 0.4, B 0.7 50–59: M 25.5, F 19.6, B 22.5 60–69: M 105.6, F 73.6, B 89.4 70–79: M 193, F 126.6, B 157.6 ≥80: M 183.6, F 99.5, B 132.4

Thugbah, Saudi Arabia (Al Rajeh et al, 1993)^28^	Not stated	Not stated	Crude rate (all ages): 0.27	Not reported

West Bengal, India (Das et al, 1996)^29^	Not stated	Not stated	Crude rate (all ages): 16.1	Not reported

West Bengal, India (Saha et al, 2003)^30^	Not stated	Not stated	Age-adjusted rate (all ages): 147 (standardized to 1990 USA population)	51–60: M 164.2, F 214.2 61–70: M 773.2, F 915.2 >70: M 552.5, F 1666.7

Kinmen, China (Wang et al, 1996)^17^	78%	67.8	Crude rate (≥50 years): M 610, F 564, B 587 Age-adjusted rate (all age): 119	50–59: M 364, F 157, B 273 60–69: M 593, F 473, B 535 70–79: M 643, F 504, B 565 ≥80: M 1923, F 1792, B 1839

Greater Beijing, China (Zhang et al, 2003)^18^	69%	68.5	Crude rate (≥55 years): M 1200, F 1100, B 1100	55–64: M 300, F 300, B 300 65–74: M 1200, F 1100, B1200 75–84: M 3800, F 3400, B 3500 85–94: M 3900, F 3600, B 3700 ≥95: M 0, F 0, B 0

Beijing, Xian, Shanghai, China (Zhang et al, 2005)^19^	48% (68% in rural areas, 37% in urban areas)	69	Crude rate (≥55 years): M 1150, F 1000, B 1070 Crude rate (≥65 years): M 1700, F 1640, B 1700 Age-adjusted rate (≥55 years): M 1410, F 1270, B 1340 Age-adjusted rate (≥65 years): M 2120, F 1980, B 2100 (Standardized to 2000 US census)	55–64: M 400, F 250, B 320 65–74: M 1210, F 1050, B 1130 75–84: M 2820, F 2670, B 2740 ≥85: M 4010, F 4040, B 4030

Taiwan (Chen et al, 2001)^31^	Not stated	Not stated	Crude rate (≥40 years): M 302.2, F 431.9, B 367.9 Age-adjusted rate (≥40 years): M 299.2, F 423.7, B 357.9 Age-adjusted rate (all ages): M 108.7, F 154.0, B 130.1 (standardized to 1970 US census)	40–49: M 78.3, F 0, B 37.8 50–59: M 252.5, F 0, B 122.5 60–69: M 224.9, F 896.5, B 546.7 70–79: M 645.2, F 1000, B 819.7 ≥80: M 2013, F 2326, B 2199

Linxian, China (Zhang et al, 2005)^20^	14%		Crude rate (≥50 years): M 614, F 459, B 522	50–59: M 177, F 56, B 103 60–69: M 726, F 555, B 621 70–79: M 912, F 894, B 902 ≥80: M 1974, F 1563, B 1744

Korea (Seo et al, 2007)^35^	Not stated	Not stated	Crude rate(>18 years): M 498, F 271, B 374	Not specified

Singapore (Tan et al, 2004)^32^	Not stated	63.7	Crude rate (≥50 years): M 360, F 230, B 290, Age-adjusted rate (≥50 years): M 310, F 200, B 250 (standardized to UICC world population), Age-adjusted rate (≥50 years): 300 (standardized to the US 1970 population)	50–59: M 110, F 0, B 50 60–69: M 340, F 240, B 280 70–79: M 590, F 450, B 510 >80: M 1420, F 1080, B 1250

Yonago, Japan (Harada et al, 1983)^22^	Not stated	64.3	Crude rate (all ages): M 63.3, F 96.6, B 80.6 Crude rate (≥50 years): 283	30–39: M 0, F 9.4, B 4.7 40–49: M 35, F 44.5, B 39.9 50–59: M 103.4, F 71.5, B 85.8 60–69: M 144.5, F 319.3, B 245.1 70–79: M 649.1, F 732.6, B 698.4 80–89: M 918.8, F 662.8, B 752.7

Izumo city, Japan (Okada et al, 1990)^23^	17%	60	Crude rate (all ages): M 46.7, F 113.8, B 82 Age-adjusted rate (all ages): 68.3 (standardized to Japanese 1985 population census)	30–39: M 0, F 47.5, B 23.2 40–49: M 20.1, F 19.1, B 19.6 50–59: M 76.6, F 51.8, B 63.6 60–69: M 308.5, F 362.1, B 338.6 70–79: M 110.5, F 717.9, B 478.7 80–89: M 133.9, F 448.4, B 335.7

Yonago, Japan (Kusumi et al, 1996)	Not stated	70.4	Crude rate (all ages): M 72.8, F 159.1, B 117.9	0–39: M 0, F 0, B 0 40–44: M 0, F 16.6, B 8.4 45–49: M 21.4, F 61.4, B 41.8 50–54: M 24.1, F 22.6, B 23.3 55–59: M 49.5, F 92.0, B 71.5 60–64: M 134.9, F 273.5, B 210.0 65–69: M 370.7, F 522.3, B 457.9 70–74: M 766.7, F 611.7, B 669.1 75–79: M 432.6, F 1107.2, B 850.5 ≥80: M 468.0, F 883.0, B 750.0

Hokkaido, Japan (Moriwaka et al, 1996)^25^	Not stated	Not stated	Hokkaido: Crude rate (all ages): 94.7 Iwamizawa: ​ Crude rate (all ages): M 91.3, F 99.8, B 95.8 ​ Age-adjusted rate (all ages): M 63.2, F 77.8, B 71.2 (standardized to the US 1970 population)	40–49: M 16.4, F 14.8, B 15.6 50–59: M 76.0, F 83.0, B 79.8 60–69: M 256.4, F 228.9, B 241.8 70–79: M 896.6, F 723.3, B 800.7 ≥80: M 0, F 554.0, B 358.4

Israel (Anca et al 2002)^40^	Not stated	66.7	Crude rate (all ages): 240 Crude rate (>40 years): 233 Crude rate (>60 years): 179 Age-adjusted rate (>40 years): 337 (the reference the rate was standardized to was not reported)	>40 years: 330 >60 years: 942 M:F ratio (all ages): 1.3:1

Yamagata, Japan (Kimura et al, 2002)^26^	Not stated	Not stated	Crude rate (all ages): 118.7 Age-adjusted rate (all ages): M 61.3, F 91, B 76.6 (standardized to 1995 Japanese census)	0–29: M 0.9, F 0, B 0.5 30–39: M 3, F 1, B 2 40–49: M 10, F 5, B 8 50–59: M 50, F 35, B 42 60–69: M 152, F 141, B 146 70–79: M 318, F 461, B 403 >80: M 332, F 345, B 341

M: male, F: female, B: both sexes.

**Table 4.** Age-standardized prevalence of Parkinson’s disease in Asian and worldwide studies reporting age-specific rates (standardized to the WHO World Population 2000)

Survey type and countries	Prevalence
Door-to-door survey	
​ Asia	
​ ​ Six cities, China^21^	51.3
​ ​ 29 provinces, China^16^	16.7
​ ​ Kinmen, China^17^	112.2
​ ​ Greater Beijing, China^18^	109.3
​ ​ Linxian, China^20^	112.2
​ ​ Beijing, Xian, Shanghai, China^19^	176.9
​ ​ Taiwan^31^	113.1
​ ​ Singapore^32^	61.9
​ ​ Bombay, India^27^	140.6
​ ​ Israel^40^	113.9
​ Worldwide	
​ ​ Sicily, Italy^41^	173.8
​ ​ France^42^	101.0
​ ​ Spain^43^	117.6
​ ​ Rotterdam, Netherlands^44^	216.0
​ ​ Europarkinson ​ (France, Italy, the Netherlands, Spain)^45^	135.9
​ ​ Argentina^46^	174.3
​ ​ Brazil^47^	297.7
​ ​ Bolivia^48^	106.5
​ ​ Sydney, Australia^49^	439.4

Record-based survey	
​ Asia	
​ ​ Yonago city, Japan^22^	68.3
​ ​ Izumo city, Japan^23^	57.9
​ ​ Yonago city, Japan^24^	68.2
​ ​ Hokkaido, Japan^25^	61.4
​ ​ Yamagata, Japan^26^	35.8
​ Worldwide	
​ ​ Ferrara, Italy^50^	95.5
​ ​ San Marino, Italy^51^	100.4
​ ​ Central Italy^52^	89.7
​ ​ Sardinia^53^	61.4
​ ​ Scotland^54^	91.4
​ ​ Northampton, England^55^	70.9
​ ​ London, England^56^	91.7
​ ​ Northampton, England^57^	73.5
​ ​ Spain^58^	93.8
​ ​ Bulgaria^59^	141.1
​ ​ Estonia^60^	111.3
​ ​ Manhattan, New York, City, USA^13^	81
​ ​ Sydney, Australia^61^	71.3

#### Incidence

**Figure 1.**
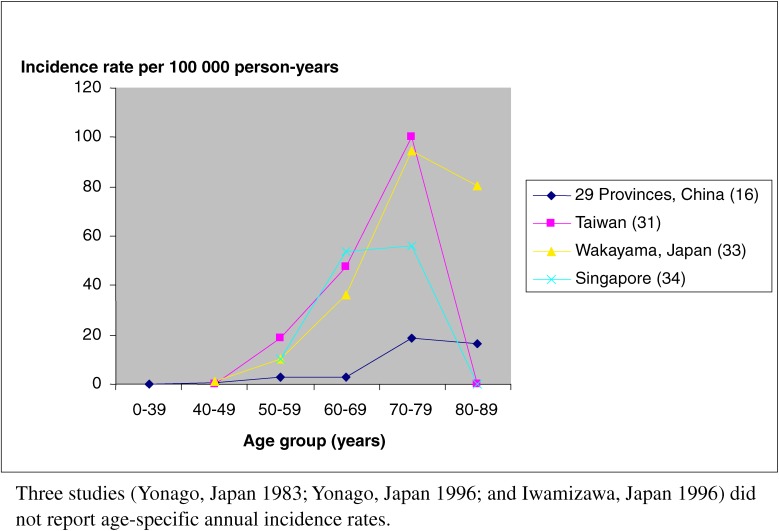
Incidence of Parkinson’s disease in Asia

Five of the 7 studies of PD incidence were record-based (Table 2). The crude incidence rates were reported differently: some reported the rate in all age groups and others reported incidence only in specific age groups. The overall crude incidence rate was 1.5, 8.7, 10.2, and 15 per 100 000 person-years^16^^,^^22^^,^^24^^,^^25^ (3 studies did not report the overall crude incidence rate^22^^,^^24^^,^^25^). The crude incidence rates were 16.9 and 30.1 among subjects 40 years or older,^31^^,^^33^ and 33 among subjects over 50 years.^34^ Figure 1 shows the incidence rate per 100 000 person years from studies that reported age-specific annual incidence rates. The incidence rates, standardized to WHO World Standard Population 2000, were 1.5 and 8.7 per 100 000 person-years in door-to-door surveys conducted for the 29-Province Study in China and in a Taiwanese study, respectively (Table 5). The same rates from the record-based surveys were 6.7 and 8.3 per 100 000 person-years in Singapore and Wakayama, Japan, respectively (Table 5). The mean age of symptom onset was 60 to 69 years; onset before the age of 50 years occurred only rarely. The age-specific incidence increased from approximately 10 to 18.5 per 100 000 person-years between the ages of 50 and 59 and rose sharply after the age 60, to between 94.5 and 100.2 per 100 000 person-years for people aged 70 to 79 years. The incidence was highest from age 70 to 79 years and declined in participants older than 80 years (Figure 1).

**Table 5.** Age-standardized incidence of Parkinson’s disease from Asian and worldwide studies reporting age-specific rates (standardized to the WHO World Population 2000)

Survey type and countries	Incidence	Male:female ratio (95% confidence interval)
Door-to-door survey		
​ Asia		
​ ​ 29 provinces, China^16^	1.5	1.1 (0.6–1.8)
​ ​ Taiwan^31^	8.7	1.1 (0.4–3.1)
​ Worldwide		
​ ​ Italy^62^	27.6	1.7 (1.0–2.7)
​ ​ Rotterdam, Netherlands^63^	20.4	1.3 (0.8–2.1)
​ ​ Spain^64^	15.4	2.4 (1.1–5.0)

Record-based survey		
​ Asia		
​ ​ Wakayama, Japan^33^	8.3	1.0 (0.7–1.3)
​ ​ Singapore^34^	6.7	1.2 (0.4–3.8)
​ Worldwide		
​ ​ Ferrara, Italy^50^	6.1	0.9 (0.7–1.1)
​ ​ Sweden^65^	6.5	1.5 (—)
​ ​ United Kingdom^66^	12.4	—
​ ​ Cambridge, UK^67^	8.2	1.1 (0.8–1.7)
​ ​ Hawaii, USA^15^	9.4	—
​ ​ Rochester, Minnesota, USA^68^	17.4	1.6 (1.3–1.9)
​ ​ Omsted, Minnesota^69^	10.3	2.0 (1.6–2.6)
​ ​ Manhattan, New York City, USA^13^	9.5	0.9 (0.6–1.3)
​ ​ ​ Black men/women	11.0	1.7 (0.7–3.7)
​ ​ ​ White men/women	8.5	0.7 (0.4–1.2)
​ ​ ​ Other men/women (includes Asian)	9.1	0.7 (0.2–2.1)
​ ​ Northern California^14^	9.8	1.7 (1.4–2.0)
​ ​ ​ Non-Hispanic white	9.9	1.7 (1.4–2.1)
​ ​ ​ Black	7.3	1.7 (0.8–3.5)
​ ​ ​ Asian	7.6	1.3 (0.7–2.5)
​ ​ ​ Hispanic/Latino	12.6	2.0 (1.1–3.6)

#### Comparison to worldwide studies

After standardization of the reported prevalence and incidence rates of PD in both Asian and non-Asian countries, both prevalence and incidence were lower in Asian populations. The age-standardized prevalence reported in door-to-door surveys ranged from 16.7 to 176.9 per 100 000 in Asian countries, and from 101.0 to 439.4 per 100 000 in non-Asian countries (Table 4). Excluding 1 study with questionably low prevalence and incidence,^16^ the standardized prevalence ranged from 51.3 to 176.9 per 100 000. In the record-based surveys, the standardized prevalence per 100 000 was 35.8 to 68.3 in Asian countries, and 61.4 to 141.1 in non-Asian countries. The 2 door-to-door surveys that reported age-specific incidence had lower standardized incidence rates, as compared to incidence reported in Western countries. The standardized incidence rates from 2 Asian record-based studies were 6.7 and 8.3 per 100 000 person-years, as compared to 6.1 to 17.4 (most were higher than 9.4) per 100 000 person-years in Western countries. The highest prevalences of PD in Asia were in China and in the Parsis population in India. However, PD prevalence in China varied considerably, from the lowest (16.7 per 100 000) to the highest (176.9 per 100 000) among Asian countries. A study in northern California showed that the age- and sex-adjusted incidence rate was highest among Hispanics, followed by non-Hispanic whites, Asians, and blacks (Table 5).^14^ The standardized incidence rate of PD in an Asian population in northern California was reported to be 7.6 per 100 000 person-years. The Honolulu Heart Study, which followed 8006 American men of Japanese or Okinawan ancestry for 29 years, reported a rate of PD incidence that was similar to that of a Japanese study (Table 5).^15^

Worldwide, the incidence rate of PD was higher among men than women, especially in older populations. Table 5 summarizes the male:female ratios reported in Asian and worldwide studies. The male:female ratios for Asian PD incidence ranged from 1.0 to 1.2, which was lower than those reported worldwide (range, 0.7–2.4). However, because the ratio ranges overlapped, we cannot conclude that there is a sex difference in PD incidence between Asian and non-Asian populations.

## DISCUSSION

Estimates of the prevalence and incidence of PD were lower in studies of Asian populations than in studies conducted in non-Asian countries. The results of prevalence studies were quite conclusive: the prevalence of PD in Asian countries was slightly lower than that of Western countries in both door-to-door surveys and record-based studies. However, the difference was more obvious in record-based studies. We speculate that the discrepancy in the prevalences observed in door-to-door surveys and record-based surveys of Asian and non-Asian countries is due to differential access to health care services. The lack of door-to-door surveys of incidence in Asian countries makes it difficult to conclude that incidence was truly lower than in Western countries, as only 2 studies reported age-specific incidence, which is necessary if results are to be standardized with the standard population. Also, 1 of these 2 studies reported a very low prevalence and incidence, as compared to other studies from the same country. However, these 2 studies reported that the incidence of PD was lower in Asian countries than in Western countries.

Differences in survival may explain variation in prevalence. The prevalence of PD is influenced by the incidence and duration of illness. The sex difference in PD in different age groups might simply reflect differences in survival once PD has developed, as well as differences in life expectancy. Record-based studies may not be generalizable to the population, as they did not include patients with mild PD, ie, those who were unlikely to seek medical treatment. The door-to-door surveys reported higher prevalences than did record-based surveys. This was also true for incidence studies in Western countries, but not those conducted in Asian countries. However, this is likely due to the fact that there were an insufficient number of incidence studies to demonstrate a difference.

Diagnosis of PD mainly relies on clinical presentation. Neurological examination is often difficult, especially in very old people. Case ascertainment in older populations may be inaccurate due to the high incidence of subtle extrapyramidal signs on neurological examination in people with no known neurological or psychiatric disease. Extrapyramidal signs may be age-related, making it difficult to separate early parkinsonism from normal aging. A study examining the accuracy of PD diagnosis in general practices showed that the diagnosis of clinically probable PD was confirmed in 53% of presumed PD cases.^36^ To ensure the accuracy of case ascertainment in epidemiological data, the diagnosis should ideally be confirmed by a neurologist, which is unfeasible in some countries.

We found that PD incidence declined after the age of 80 years in Asian countries, but increased in other regions of the world. Advancing age is associated with an increase in the incidence of PD, but it may also contribute to differences in clinical presentation. Patients with late-onset PD have greater motor impairment than do patients with middle-age onset.^37^ This might be due to more rapid disease progression, less aggressive or less potent medical treatment, or the effects of comorbid illnesses. One door-to-door survey found that the majority of undiagnosed PD patients were aged 80 years or older.^38^ Lower incidence in older age may therefore be due to underdiagnosis, restricted access to health service, and/or difficulty of diagnosis resulting from comorbidity. Incidence rates in the very old may also be inaccurate because estimates are based on small numbers of cases in this age group.

Epidemiological data show that the prevalence rate of PD is higher in whites than in Hispanics, blacks, and Asians.^13^ However, this finding remains controversial. Possible explanations for the discrepancy include variation in population characteristics, case definitions, methods of case ascertainment, sources of PD cases, and denominator populations. Once standardized to the WHO World Standard population, PD incidence among Asian populations in Western countries is similar to that in Asian countries (Table 5). Racial differences in prevalence are most likely due to variations in age distribution. A Singaporean study observed similar age-adjusted prevalence rates of PD among Chinese, Malays, and Indians in Singapore.^32^ However, the participation rate for this study was 64% and there were discrepancies between population proportions and participation rates. In addition, the incidence rates differed among these 3 ethnic groups. The differences could result from the small numbers of cases or from the different rates of utilization of hospital services.

To our knowledge, this is the first systematic review of the epidemiology of PD in Asian populations. The main limitation of this review is the small number of studies conducted in Asia. In addition, many studies did not report age-specific prevalence. The problems that are common to all PD epidemiological studies and can affect the interpretation of the results are summarized below.

### Case ascertainment and variation in diagnostic criteria

For ease of comparison, the same criteria should be used for case ascertainment in each study. Different diagnostic criteria influence prevalence estimates; therefore, comparison of surveys that use different diagnostic criteria leads to imprecision. In addition, inclusion and exclusion criteria vary. Earlier studies tended to use less specific criteria than more recent studies. Stricter criteria result in higher specificity, but lower sensitivity; broader criteria have the opposite effect. Determination of the criteria used to define PD must account for the delicate balance between sensitivity and specificity.

### Variation in presentation

Because PD is a chronic disease, the point of reference for defining an incident case would ideally be symptom-based. Referring to the date of diagnosis is undesirable because it depends on access to health care, which often differs by country.

### Sampling bias in the selection of participants

Door-to-door surveys may result in unrepresentative samples of the parent population if there is a low response rate. Therefore, every study should report the response rate. People who did not participate might have parkinsonism that prevents them from participating in the studies, or they may be from a group with a low prevalence of PD. Studies with a low participation rate are therefore more subject to imprecision. The studies in China, Japan, and Saudi Arabia had high participation rates, which are not common in many Western countries because of legal constraints and concerns about privacy. Case ascertainment methods included the use of hospital-based records, door-to-door surveys, and self-reporting. Even in the door-to-door surveys, there was considerable variation in the techniques (eg, 1-stage and 2-stage surveys), instruments, identified informants, and interviewers. These differences definitely affect the outcomes of the studies. Record-based studies may underestimate incidence and prevalence, especially in countries with restricted access to health care. Because PD symptoms are chronic and range from mild to severe, the tendency to seek medical care and the availability of medical service may cause differences in estimates of PD prevalence and incidence. For example, the rural-urban difference in the incidence of PD may simply be due to differential access to health care services. Moreover, the distribution of PD may be distorted, as there might be a greater number of atypical cases in hospital-based records (ie, young-onset cases) than in the general population.

### Error and bias in numerator data

Interobserver variation (observer variation) in asking questions and performing neurological examinations should be taken into account and, when possible, the number of observers used should be kept to a minimum. The observers should be properly trained in administering the test or phrasing questions. PD symptoms can fluctuate and may be difficult to detect in mild cases (subject variation). Any study that depends on patient assessment at 1 time point may include misclassified cases.^39^ The diagnosis of early PD is difficult and sometimes requires long-term follow-up to monitor responses to dopaminergic therapy, disease progression, and the development of any features suggestive of parkinsonian-plus syndrome.^3^ Also, patients with a diagnosis of drug-induced parkinsonism may actually have concomitant PD. A diagnosis of PD can then only be made after administration of the drugs in question is stopped and patients are reassessed. One study reported a false positive rate of 8% and a false negative rate of 9% on a second examination 2 months after preliminary diagnosis.^19^ Hence, if possible, assessment of cases should be conducted at more than 1 time point. However, long-term follow-up is time consuming and expensive and may be impractical in population-based studies. The questionnaires and instruments involved in door-to-door surveys should be therefore be based on a single standard. Personnel should be trained according to a set curriculum. Moreover, the actual number of PD cases, the population, and the age-specific rates should be reported.

### Error and bias in the denominator

In different studies, age strata were represented by different age bands. Age-specific rates should be presented as standard 10-year age bands, which would make data more comparable. The prevalence and incidence rates should be standardized to the worldwide standard, as well as to the national or continental standard. Though PD is a common neurodegenerative disease, it is still relatively uncommon when compared to many other diseases. Therefore, a large sample size is required to yield statistically accurate estimates.

### Limitations

A limitation of this review is potential publication bias. Studies from Asian countries might be published only in domestic and non-English language journals, which may not be easily accessible. However, a study published in a domestic journal is more likely to be small and to provide imprecise estimates of prevalence and incidence. Another limitation of this review is the different inclusion and exclusion criteria used in selecting the studies. The criteria used for worldwide studies were more stringent. However, as the primary objective of this review was to analyze the prevalence and incidence rates of PD in Asia, and there are a great number of studies worldwide, we had to limit the inclusion of studies to ensure a high standard for the worldwide studies.

### Conclusion and recommendations

In the future, most new PD cases are likely to be in Asia, due to the growing number of older people in the region. However, there have been few descriptive epidemiological studies conducted in Asian countries. The prevalence of PD in Asian populations is slightly lower than in the Western world, although it is difficult to compare incidence as there have been only a small number of studies. The lack of well-designed large-scale studies limits our understanding of the epidemiology of PD. Varying methodologies, diagnostic criteria, and case-finding strategies all contribute to the variation in the reported prevalence and incidence of PD. In the future, it would be highly desirable to conduct a large prospective study, with a 2-phase door-to-door survey methodology, identical diagnostic criteria, participants examined by specialists more than once, identical age strata, and age-standardization rates calculated to a reference population. Such a study would ideally encompass several divergent countries within Asia, to produce estimates that are more comparable and precise.

## ONLINE ONLY MATERIALS

eAppendix.
