# Supplementary material for: Systematic Review of the Prevalence and Incidence of Parkinson’s Disease in Asia
Source: J Epidemiol. 2009 Nov 5;19(6):281–93. doi: 10.2188/jea.JE20081034 (PMC3924097; doi:10.2188/jea.JE20081034)
Supplement: eAppendix. [file je-19-281-s001.pdf]

Supplementary Data

Appendix 1. Prevalence studies of Parkinson's disease (by region)

Study	Study period	Design	Case finding	Diagnostic criteria	Age of cases	Percent examined by specialists	Population	PD cases
Ferrara, Italy[1]	1967-1987	Record-based study	Medical records and prescriptions from hospitals, neurologists, national health insurance scheme, nursing home, general practitioners	Deterioration in neurological function in patients with $\geq$2 of 4 cardinal signs	$\geq$ 35	32%	187 381	291
San Marino, Italy[2]	1985-6	Record-based study	Medical records from hospitals, general practitioners, pharmacies	Deterioration in neurological function in patients with $\geq$2 of 4 cardinal signs and exclusion of secondary causes	All ages	97%	22 322	34
Sicily, Italy[3]	1987	Door-to-door survey	2 phases door-to-door survey, questionnaire, neurological examination	$\geq$2 of 4 cardinal signs, if not receiving antiparkinsonian drugs, Or $\geq$1 of 4 cardinal signs, if improved by medications and exclusion of secondary causes	All ages	100%	24 496	63
Central Italy[4]	2001	Record-based study	Medical records from hospitals, general practitioners, nursing home and pharmacy records	$\geq$ 2 of 4 cardinal signs plus exclusion of other causes	All ages	Not stated	297 424	682

Study	Study period	Design	Case finding	Diagnostic criteria	Age of cases	Percent examined by specialists	Population	PD cases
Sardinia[5]	1972-4	Record-based study	Medical records from hospitals, physicians, National Health Insurance, National Pension Institute	≥ 2 of 4 cardinal signs plus exclusion of arteriosclerotic cause	≥30	92.5%	1 473 800	967
Northampton, England[6]	1982	Record-based study	General practitioners, hospital consultants	Akinetic-rigid syndrome (Webster scale)	All ages	Not stated	208 000	223
Scotland[7]	1983-1984	Record-based study	Medical records from hospitals, general practitioners	>2 of 4 cardinal signs	≥ 40	38%	151 616	249
Northampton, England[8]	1991-3	Record-based study	General practitioners, hospital consultants	PDS Brain Tissue Bank criteria	All ages	92.5%	302 000	384
London, England[9]	1997	Record-based study, follow-up at 1 year	Computerized records, Linkage scheme between the National Hospital of Neurology and Neurosurgery and general practitioners	UK Brain Bank Criteria	≥50	Not stated	121 608	156
France[10]	1988-9	Door-to-door survey	2-phase door-to-door survey, questionnaire, and neurological examination	≥ 2 of 4 cardinal signs plus exclusion of other causes	≥65	Household sample: 54% Institutional sample: 100%	3 149	60
Spain[11]	1994	Door-to-door survey	2-phase door-to-door survey, questionnaire, and neurological examination	≥ 2 of 4 cardinal signs if not receiving antiparkinsonian drugs, Or ≥ 1 of 4 cardinal signs, if improved by medications, and exclusion of secondary causes	≥65	65.9%	5 278	81

Study	Study period	Design	Case finding	Diagnostic criteria	Age of cases	Percent examined by specialists	Population	PD cases
Spain[12]	1987-95	Record-based study	Medical records, physicians, and retirement homes	UK Brain Bank Criteria	All ages	84%	60 724	134
Rotterdam[13]	1990-1993	Door-to-door survey	2 phases: door-to-door survey, questionnaire and neurological examination	$\geq$ 2 of 4 cardinal signs if not receiving antiparkinsonian drugs Or $\geq$ 1 of 4 cardinal signs if improved by medications And exclusion of secondary causes	$\geq$ 55	100%	6 969	97
Bulgaria[14]	1999	Record-based study	Medical records	$\geq$ 2 of 4 cardinal signs And exclusion of secondary causes	All ages	100%	119 910	202
Europarkinson collaborative study[15] (France, Italy, Netherlands, Spain)	1988-1994	Door-to-door survey	2 phases: door-to-door survey, questionnaire and neurological examination	$\geq$ 2 of 4 cardinal signs if not receiving antiparkinsonian drugs Or $\geq$ 1 of 4 cardinal signs if improved by medications And exclusion of secondary causes	$\geq$ 65	100%	14 636	320
Estonia[16]	1986-1996	Record-based study	Medical records from a hospital, general practitioners, family doctors, and nursing home	UK Brain Bank Criteria	All ages	100%	153 240	270

Study	Study period	Design	Case finding	Diagnostic criteria	Age of cases	Percent examined by specialists	Population	PD cases
Manhattan, USA[17]	1988-1993	Record-based study	Medical records from hospitals, private practitioners, nursing homes and other health-related facilities, local health maintenance organizations; interview and examination by neurologists	1) Bradykinesia plus ≥1 of other 3 cardinal signs<br>2) ≥ 3 of<br>- unilateral onset<br>- persistence of asymmetry<br>- improvement by levodopa<br>- progressive course<br>- levodopa-induced chorea	All ages	100%	213 302	228
Argentina[18]	1991	Door-to-door survey	2-phase door-to-door survey: questionnaire, then examined by specialists	≥ 2 of 4 cardinal signs plus exclusion of other causes	≥40	100%	7 765	51
Brazil[19]	1997-2001	Door-to-door survey	2 phases: door-to-door survey, questionnaire to all residents ≥64 years on census, then examined by specialists	UK Brain Bank Criteria	≥ 64	100%	1 186	39
Bolivia[20]	1994	Door-to-door survey	2 phases: door-to-door survey of random cluster of 20% of population, questionnaire and neurological examination	≥ 2 of 4 cardinal signs, at least 1 must be resting tremor or bradykinesia, exclude secondary causes	≥ 40	90%	9 955	5

Study	Study period	Design	Case finding	Diagnostic criteria	Age of cases	Percent examined by specialists	Population	PD cases
Sydney, Australia[21]	1997-9	Sample from existing population-based cohort study	Participants of the Blue Mountains Eye Study	Physician's diagnosis and clinical feature of PD: tremor, rigidity, bradykinesia, poor righting reflex	$\geq$ 49	100%	3 509	16
Sydney, Australia[22]	1998-1999 2002-2003 Pooled data from 2 studies (same methodology)	Door-to-door survey	2 phases: door-to-door survey, questionnaire and neurological examination	$\geq$ 2 of resting tremor, bradykinesia, and rigidity plus exclusion of other causes	$\geq$ 55	100%	1 028	36

Appendix 2. Incidence studies of Parkinson's disease (by region)

Study	Year	Design	Case finding	Diagnostic criteria	Percent examined by neurologists	Population	PD cases
Ferrara, Italy[1]	1967-1987	Record-based study	Medical records and prescriptions from hospitals, neurologists, national health insurance scheme, nursing home, general practitioners	Deterioration in neurological function in patients with ≥2 of 4 cardinal signs	32%	187 381	394
Italy[23]	1992-1996	Door-to-door survey with follow-up of 3.9 years	2 phases: door-to-door survey, questionnaire; if positive, examined by neurologists	≥2 of 4 cardinal signs if not receiving antiparkinsonian drugs; Or, ≥1 of 4 cardinal signs if improved by medications	100%	5 632	42
Sweden[24]	1986-1988	Record-based study	Patients/prescription reviews, examination by neurologists if not previously seen by expert	All of: 1)≥ 1 of: tremor, rigidity, hypokinesia 2) insidious progression 3) no history of neuroleptics 4) no atypical signs 5) response to levodopa	>50%, but no exact number stated	147 777	49
Rotterdam, Netherlands[25]	1990-1999	Door-to-door survey, with follow-up period of 5.8 years	2 phases: door-to-door survey 1) screen for signs of parkinsonism, method not stated 2) clinical examination by physicians specializing in neurologic disorders	>2 of 4 cardinal signs if not receiving antiparkinsonian drugs; Or, > 1 of 4 cardinal signs if improved by medications, and exclusion of secondary causes	100%	6 839	67

Study	Year	Study design,	Case findings	Diagnostic criteria	Percent examined by neurologists	Population	PD cases
United Kingdom[26]	1995-1996	Record-based study	General practitioner clinics with linkage to a referral hospital	Not stated	100%	100 230	number not stated
Cambridge, UK[27]	2000	Record-based study from primary and secondary care	Referral from GP, neurologists, geriatricians, old-age psychiatrists, PD specialist nurse, hospital discharge coding departments	UK Brain Bank Criteria	77%	708 715	201
Spain[28]	1997-1998	Door-to-door survey	Population aged $\geq$65 yrs on census of specified areas; 2 phases: door-to-door survey, questionnaire: if positive, clinical examination	$\geq$2 of 4 cardinal signs if not receiving antiparkinsonian drugs; Or, $\geq$1 of 4 cardinal signs if improved by medications; and exclude other causes	100%	5 160	30
Hawaii[29]	1965-1994	Record-based study	Honolulu Heart Study<br>- medical records (hospitals, neurologists' offices)<br>- death certificates<br>- rescreen with standardized questions and physical examination	> 2 cardinal signs plus clinical response to levodopa	>50%, actual figure not stated	8 006	92

Study	Year	Study design,	Case findings	Diagnostic criteria	Percent examined by neurologists	Population	PD cases
Rochester, USA[30]	1967-1979	Record-based study	Medical records from general practitioners, specialty clinic in hospitals	Any of<br>1) diagnosis of parkinsonism by neurologists<br>2) all 3 of resting tremor, bradykinesia, rigidity<br>3) exaggerated glabellar reflex, reduced facial expression, unilateral or asymmetrical bradykinesia and rigidity<br>4) positive histological findings	0%	Not stated	118
Olmsted, Minnesota[31]	1976-1990	Record-based study	Medical records of the Rochester Epidemiology Project	$\geq$2 of 4 cardinal signs, with<br>1) no secondary causes<br>2) levodopa responsive<br>3) no prominent or early signs of more extensive nervous system involvement	0%	95 000	154
Manhattan, USA[17]	1988-1993	Record-based study, 85% of cases received at least 1 annual follow-up	Medical records from hospitals, private practitioners, nursing homes, and other health-related facilities, local health maintenance organizations; interview and examination by neurologists	1) Bradykinesia plus >1 of other 3 cardinal signs<br>2) >3 of<br>- unilateral onset<br>- persistence of asymmetry<br>- improvement by levodopa<br>- progressive course<br>- levodopa-induced chorea	100%	213 000	83
Northern California[32]	1994-1995	Record-based study	Computerized databases of members of 1 health maintenance organization	1) >2 of 4 cardinal signs, at least 1 must be either resting tremor or bradykinesia<br>2) No suggestion of another cause<br>3) No atypical features	91%	Not stated	588

Appendix 3. Prevalence of Parkinson's disease (per 100 000 population)

Studies	%previously undiagnosed	Age at onset	Crude prevalence	Age-specific prevalence
Ferrara, Italy[1]	Not stated	62.6	Crude rate (all ages): 164.75<br>Age-adjusted rate: 130.4 (standardized to Italian population)	35-39: 42.78<br>40-49: 103.89<br>50-59: 186.80<br>60-69: 381.52<br>70-79: 624.31<br>80-89: 783.17
San Marino, Italy[2]	Not stated	66.4	Crude rate (all ages): 152<br>Age-adjusted rate (all ages): 185 (standardized to 1982 Emilia-Romagna population)	0-54: 0<br>55-59: 80.3<br>60-64: 380.2<br>65-69: 573.3<br>70-74: 1236.0<br>75-79: 1950.3<br>80-84: 949.3<br>≥85: 0
Sicily, Italy[3]	34.9% (men, 29.6%; women, 38.9%)	Not stated	Crude rate (all ages): 257.2<br>Crude rate (≥40 years): 651.9	0-49: 0<br>50-59: 115.6<br>60-69: 621.4<br>70-79: 1978.3<br>80-89: 3055<br>90-99: 0
Central Italy[4]	Not stated	Not stated	Crude rate (all ages): 229.3<br>Age-adjusted rate (all ages): 163.6 (standardized to 1996 European population)	0-34: 0<br>35-44: 6.7<br>45-54: 49.1<br>55-64: 145.2<br>65-74: 563.7<br>75-84: 1289.3<br>≥85: 1705.5
Sardinia[5]	Not stated	55.8	Crude rate (all ages): 65.6<br>Crude rate (≥50 years): 262	30-39: 3.3<br>40-49: 38.6<br>50-59: 204.5<br>60-69: 342.1<br>70-79: 311.3<br>80-89: 82.6
Scotland[7]	Not stated	65.3	Crude rate (all ages): 164.2	40-44: 12.5<br>45-49: 76.1<br>50-54: 82.6<br>55-59: 72.6<br>60-64: 239.8<br>65-69: 268.5<br>70-74: 707.4<br>75-79: 1019.6<br>80-84: 1792.1<br>≥ 85: 2205.3
Northampton, England[6]	Not stated	Not stated	Crude rate (all ages): 108	<50: 3<br>50-59: 64<br>60-69: 277<br>70-79: 702<br>80-99: 1136

Studies	%previously undiagnosed	Age at onset	Crude prevalence	Age-specific prevalence
London, England[9]	Not stated	Not stated	Crude rate (all ages): 128<br>Age-adjusted rate: 168<br>(standardized to 1997 UK population)	0-29: 0<br>30-39: 8<br>40-49: 12<br>50-59: 109<br>60-69: 342<br>70-79: 961<br>≥ 80: 1 265
Northampton, England[8]	Not stated	Not stated	Crude rate (all ages): 121	<40: 0.6<br>40-44: 4<br>45-49: 10<br>50-54: 76<br>55-59: 111<br>60-64: 159<br>65-69: 343<br>70-74: 664<br>75-79: 856<br>80-84: 1 400<br>≥ 85: 1044
France[10]	5%	Not stated	Crude rate (≥ 65 years): 1 400	
Spain[11]	28.4%	70	Crude rate (≥ 65 years):1 500	65-69: 500<br>70-74: 1 600<br>75-79: 1 900<br>80-84: 3 200<br>≥ 85: 1 500
Spain[12]	Not stated	63.6	Crude rate (all ages): 220.6<br>Age-adjusted rate: 121.9<br>(standardized to European standard population)	0-39: 3.3<br>40-49: 16.5<br>50-59: 100.2<br>60-69: 435.6<br>70-79: 953.3<br>80-89: 973.1<br>≥90: 263.1
Rotterdam[13]	12%	Not stated	Crude rate (≥ 55 years): 1 400	55-64: 300<br>65-74: 1200<br>75-84: 4200<br>85-94: 4300
Bulgaria[14]	Not stated	67.9	Crude rate (all ages): 168.5<br>Crude rate (≥50 years): 558.3<br>(standardized to Bulgarian standard population)	0-49: 0<br>50-54: 45.4<br>55-59: 100.9<br>60-64: 233.1<br>65-69: 541.8<br>70-74: 918.7<br>75-79: 2358.8<br>≥80: 3456.2
Europarkinson[15]	24%, varied from 11% in France, 13% in Netherlands, 26% in Girona, Spain and 52% in Pamplona, Spain	Not stated	Crude rate (≥ 65 years): 2 006<br>Age-adjusted (≥ 65 years): 1 600<br>(standardized to 1991 European population)	65-69: 625<br>70-74: 998<br>75-79: 2736<br>80-84: 3556<br>85-89: 3538<br>90-94: 2521<br>95-99: 625

Studies	%previously undiagnosed	Age at onset	Crude prevalence	Age-specific prevalence
Estonia[16]	Not stated	66.9	Crude rate (all ages): 176<br>Age-adjusted (all ages): 152<br>(standardized to Estonian general population)	0-39: 0<br>40-49:22<br>50-59: 127<br>60-69: 493<br>70-79: 1 232<br>≥80: 1 109
Manhattan, USA[17]	Not stated	65.7	Crude rate (all ages): 106.9	<45: 1.3<br>45-64: 99.3<br>65-74: 509.5<br>75-84: 1 192.9<br>≥ 85: 823.8
Argentina[18]	23.5%	64	Crude rate (≥40 years): 656.8	40-49: 0<br>50-59: 152.9<br>60-69: 636.9<br>70-79: 1727<br>≥80: 3385.4
Brazil[19]	72%	Not stated	Crude rate (≥ 65 years): 3 300	64-69: 800<br>70-74: 2 900<br>75-79: 2 800<br>80-84: 8 500<br>≥ 85: 14 300
Bolivia[20]	Not stated	Not stated	Crude rate (all ages): 50.2,<br>Crude rate (≥ 40 years): 286,<br>Age-adjusted rate (all ages): 97.3,<br>Age-adjusted rate (≥ 40): 304<br>(Standardized rate to world standard population)	40-49: 133<br>50-59: 371<br>≥ 60: 443
Sydney, Australia[21]	Not stated	Not stated	Crude rate (≥ 49 years): 460<br>Age-adjusted rate (all ages): 104<br>Age-adjusted rate (≥ 50): 362<br>(Standardized rate to 2001 Australian population)	49-59: 0<br>60-69: 480<br>70-79: 820<br>≥80: 560
Sydney, Australia[22]	Not stated	Not stated	Crude rate (≥55 years): 780	55-59: 1 639<br>60-69: 1 159<br>70-79: 3 951<br>≥ 80: 9 091

Appendix 4. Incidence of Parkinson's disease (per 100 000 person years)

Study	PD cases	Crude/<br>age-adjusted incidence rate	Age-specific annual<br>incidence rate
Ferrara, Italy[1]	394	Crude rate (all ages): 10.01<br>Age-adjusted rate (all ages): 8.55<br>(standardized to Italian population)	35-39: 1.14<br>40-44: 4.35<br>45-49: 6.90<br>50-54: 13.91<br>55-59: 20.99<br>60-64: 43.73<br>65-69: 29.28<br>70-74: 32.51<br>≥ 75: 13.14
Italy[23]	42	Crude rate (65-84 years): 346<br>Age-adjusted rate (65-84 years): 326<br>(standardized to Italian population 1992)	65-69: 221<br>70-74: 239<br>75-79: 353<br>80-84: 678
Sweden[24]	49	Crude rate (all ages): 11<br>Age-adjusted rate (all ages): 7.9<br>(standardized to European standard population)	30-39: 1.6<br>40-49: 3.3<br>50-59: 9.0<br>60-69: 22.4<br>70-79: 59.4<br>80-89: 79.5
Rotterdam, Netherlands[25]	67	Crude rate in total population: not stated<br>Crude rate (55-85 years): 170	55-65: 30<br>65-75: 140<br>75-85: 330<br>≥ 85: 430
United Kingdom[26]	number not stated	Crude rate: not stated<br>Age-adjusted rate (all ages): 19	45-49: 20<br>60-64: 50<br>65-69: 37<br>70-74: 222<br>75-79: 100<br>≥ 80: 116
Cambridge, UK[27]	201	Crude rate (all ages): 13.6	30-39: 0.9<br>40-49: 2.0<br>50-59: 9.6<br>60-69: 41.2<br>70-79: 75.5<br>≥ 80: 86.2
Spain[28]	30	Crude rate (≥ 65-85 years): 235.9<br>Age-adjusted rate (≥ 65-85 years): 186.8<br>(standardized to European standard population)	65-69: 67.5<br>70-74: 225.3<br>75-79: 222.1<br>80-84: 298.8<br>≥ 85: 365.9

Study	PD cases	Crude/	Age-specific annual

		age-adjusted incidence rate	incidence rate
Hawaii[29]	92	Crude rate (≥ 45-94 years): 53.1<br>Age-adjusted rate (all ages): 11.1<br>Age-adjusted rate (≥ 50 years): 45.2<br>(standardized to 1970 US population)	45-49: 0<br>50-54: 6.1<br>55-59: 33.8<br>60-64: 27.6<br>65-69: 45.4<br>70-74: 67<br>75-79: 139.1<br>80-84: 116.9<br>85-89: 84.2<br>90-94: 0
Rochester, USA[30]	118	Age-adjusted rate (all ages):18.2<br>(standardized to 1970 US population)[a]	0-29: 0<br>30-54: 5.3<br>55-64: 32.2<br>65-74: 113.5<br>75-84: 254.4<br>≥ 85: 155.4
Olmsted, Minnesota[31]	154	Crude rate (all ages): 10.8	0-29: 0<br>30-49: 1.3<br>50-59: 17.4<br>60-69: 52.5<br>70-79: 93.1<br>80-99: 79.1
Manhattan, USA[17]	83	Crude rate (all age): 13	<45: 0<br>45-64:10.7<br>65-74: 54.2<br>75-84: 132.6<br>≥ 85: 212.8
Northern California[32]	588	Crude rate (all ages): 12.3<br>Crude rate (≥50 years): 44<br>Age-adjusted rate: 13.4<br>(standardized to 1990 US population)	<30:0<br>30-39:0.5<br>40-49:2.5<br>50-59:9.8<br>60-69: 38.8<br>70-79: 107.2<br>80-89:119

[a] Calculated as patients with parkinsonism and without drug-induced PD. PD represented 92% of incident cases.
